# Hypophosphatemia in the Diagnosis and Management of Primary Hyperparathyroidism

**DOI:** 10.3390/jcm14197024

**Published:** 2025-10-03

**Authors:** Rosario Paloma Cano-Mármol, Inmaculada Ros-Madrid, María Carmen Andreo-López, Manuel Muñoz-Torres

**Affiliations:** 1 Department of Endocrinology and Nutrition, Virgen de la Arrixaca University Clinical Hospital, 30120 Murcia, Spain; rosariopaloma.cano@um.es (R.P.C.-M.); inmarosmadrid@gmail.com (I.R.-M.); 2 Pascual Parrilla Murcian Institute of Biosanitary Research (IMIB), 30120 Murcia, Spain; 3 Department of Endocrinology and Nutrition, San Cecilio University Clinical Hospital, 18016 Granada, Spain; 4 Granada Biosanitary Research Institute (Ibs. Granada), 18012 Granada, Spain; 5 Biomedical Research Network Centre (CIBER), 28029 Madrid, Spain; 6 Department of Medicine, University of Granada, 18012 Granada, Spain

**Keywords:** hypophosphatemia, primary hyperparathyroidism, FGF-23, parathyroid adenoma, diagnosis

## Abstract

**Background**: Hypophosphatemia is a frequently underestimated metabolic disorder, yet it can be one of the first biochemical findings in primary hyperparathyroidism (PHPT). Current diagnostic and surgical criteria for PHPT do not include serum phosphate, despite its potential value as an early marker. **Methods**: We report the case of a 79-year-old woman with type 2 diabetes mellitus, hypertension and osteoarthritis, followed since 2015 for persistent hypophosphatemia (0.8 mg/dL) and stress fractures. **Results**: Initial calcium and vitamin D levels were normal, but PTH was elevated. Bone scintigraphy revealed multiple stress fractures, while ultrasound and sestamibi scan were inconclusive. Despite cholecalciferol and calcitriol supplementation, hypophosphatemia persisted. From 2023, progressive hypercalcemia developed (10.9 mg/dL), with sustained hypophosphatemia (1.7 mg/dL), persistently high PTH (121 pg/mL) and markedly elevated FGF-23 (1694 kRU/L). Renal phosphate wasting was demonstrated, with reduced tubular reabsorption. An 18F-fluorocholine PET-CT performed in 2024 identified two right parathyroid adenomas, establishing the diagnosis of PHPT. The patient was referred for parathyroidectomy. **Conclusions**: Hypophosphatemia may serve as a complementary biomarker in the diagnostic and therapeutic approach to PHPT, but only after other potential causes of low phosphate levels have been excluded, as illustrated in this case. Its consideration could facilitate the early identification of PHPT and improve clinical decision-making, particularly in patients who do not meet classical surgical indications.

## 1. Introduction

The classic definition of primary hyperparathyroidism (PHPT) is the presence of hypercalcemia associated with elevated or inappropriately normal parathyroid hormone (PTH) levels confirmed in at least two measurements taken at an interval of no less than two weeks [1]. In most cases, hypercalcemia is asymptomatic and detected incidentally on routine laboratory testing [2,3].

The most common etiology of sporadic PHPT is a single parathyroid adenoma (85–90%), followed to a lesser extent by multiglandular disease (approximately 15%) and, in rare cases, parathyroid carcinoma (<1%) [1,4]. The average age of onset is around 50 years, with a higher prevalence in women [1].

In addition to hypercalcemia, other biochemical findings suggestive of excessive PTH secretion include hypercalciuria and hypophosphatemia [2,3]. However, no existing clinical guidelines incorporate serum phosphate cut-off points among the diagnostic or surgical criteria for PHPT [5]. Although not currently included among the established diagnostic or surgical criteria, hypophosphatemia may serve a useful marker in the diagnostic workup of PHPT, particularly in patients with asymptomatic forms or non-classical phenotypes. In this context, we present a clinical case that illustrates the diagnostic value of hypophosphatemia in PHPT, highlighting its potential role as a complementary marker in the biochemical characterization of the disease.

## 2. Case Report

A 79-year-old woman under follow-up at the Endocrinology Department since 2015 for chronic hypophosphatemia and stress fractures in the context of suspected osteomalacia. Her medical history includes type 2 diabetes mellitus, hypertension, dyslipidemia, obesity, osteoarthritis, anxiety disorder and prior surgical procedures including cholecystectomy, hysterectomy and appendectomy.

Initial laboratory tests in 2015 revealed a serum phosphorus level of 0.8 mg/dL (reference range, NR: 2.5–4.5 mg/dL), with normal levels of calcium (9.8 mg/dL; NR: 8.5–10.2 mg/dL) and 25(OH) hydroxyvitamin D (25(OH)D) (28.5 ng/mL; NR: 20–40 ng/mL), alongside elevated PTH (169 pg/mL; NR: 10–65 pg/mL). Bone scan showed multiple areas of increased uptake consistent with stress fractures involving the ribs, sacroiliac joints, thoracolumbar spine and pelvis.

Cervical ultrasound identified two thyroid nodules measuring 11 and 15 mm (classified as TIRADS 2) and fine needle aspiration confirmed their benign nature. Technetium-99m sestamibi (MIBI) scan was initially inconclusive, describing hypertrophy of the right thyroid lobe with uptake in its lower pole, but without definitive findings of hyperfunctioning parathyroid tissue.

During follow-up, hypophosphatemia persisted despite supplementation with cholecalciferol and calcitriol. From 2023 onwards, serum calcium levels progressively increased, ultimately reaching elevated levels. Laboratory tests showed hypercalcemia (10.9 mg/dL), sustained hypophosphatemia (1.7 mg/dL), and persistently elevated of PTH (121 pg/mL). Estimated glomerular filtration rate was 62 mL/min and fibroblast growth factor 23 (FGF-23) was markedly elevated at 1694 kRU/L (NR: 26–110 kRU/L). Evidence of renal phosphate wasting was observed, with tubular reabsorption values below 75% (normal: 85–95%) (Table 1).

An 18F-fluorocholine PET-CT (18F-choline PET-CT) scan performed in November 2024 identified two right parathyroid adenomas, located in the upper and lower poles. The patient did not report renal colic, falls or new fractures. At the most recent follow-up, a diagnosis of PHPT was confirmed, characterized by hypercalcemia, persistent hypophosphatemia, elevated PTH and FGF-23 levels and absence of hypercalciuria or overt hyperphosphaturia.

**Figure 1:**
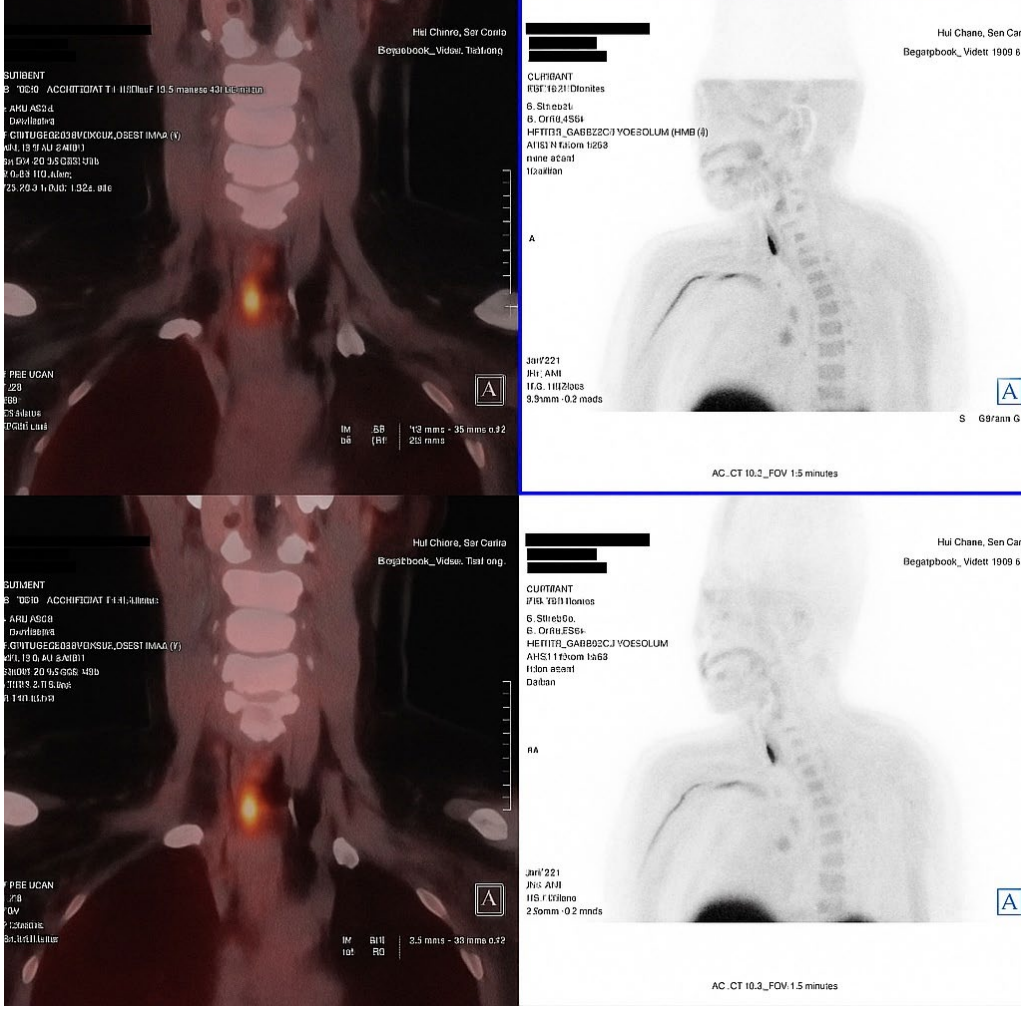
Fused coronal and axial images (**left**) and anterior projection MIP sections (**right**) identify two areas of focal increased uptake in the right cervical region. The uptake corresponds to a posterosuperior nodular lesion and an inferoposterior nodular lesion adjacent to the right thyroid lobe, findings consistent with double right parathyroid adenomas (superior and inferior). This technique localized the parathyroid adenomas, eliminating the need for FDG-PET to exclude oncogenic osteomalacia.

Secondary causes of hyperparathyroidism, including vitamin D deficiency, chronic kidney disease, and malabsorption, were excluded, thereby supporting the diagnosis of primary hyperparathyroidism (PHPT) (Figure 1).

In this case, hypophosphatemia was an early biochemical finding that persisted for nearly a decade, preceding the development of the classic biochemical profile of primary hyperparathyroidism. The patient was referred for surgery.

## 3. Discussion

Hypophosphatemia is a frequent but often underestimated mineral metabolism disorder. In the context of PHPT, it may precede the development of hypercalcemia, constituting an early and persistent biochemical abnormality [4,5]. Its clinical relevance lies in the fact that phosphorus is involved in numerous physiological processes: it constitutes a structural component of cell membranes and nucleic acids and intervenes in obtaining energy through the phosphorylation of adenosine diphosphate (ADP). In addition, it contributes to the regulation of the acid–base balance of body fluids and is essential for adequate bone mineralization [5,6,7].

Hypophosphatemia, defined as serum phosphate below 2.5 mg/dL, is classified as mild (1.8–2.5 mg/dL), moderate (1.0–1.7 mg/dL), or severe (≤0.9 mg/dL) [7]. In children, normal levels are higher due to increased growth demands [8]. The mechanisms underlying hypophosphatemia are multifactorial and include alterations in tubular phosphate handling and regulatory hormone levels [7,9].

Despite this classification, no diagnostic cut-off value has been proposed in the literature for hypophosphatemia in PHPT, largely because serum phosphate varies according to age, sex, and hormonal influences [7]. Current clinical guidelines do not incorporate phosphate as a diagnostic criterion for PHPT [1,5]. Our case illustrates that hypophosphatemia, although not a guideline-based parameter, may represent an early biochemical marker in selected patients.

Puente et al. reported a high prevalence of hypophosphatemia in diverse clinical contexts: 100% following hepatic lobectomy, 80% in sepsis, 75% in polytrauma, 29–34% in surgical intensive care patients and 22% in those with chronic obstructive pulmonary disease [10]. Furthermore, intravenous iron (carboxymaltose) or insulin therapies have been linked to moderate hypophosphatemia [10]. These data underscore the importance of systematically monitoring serum phosphate in critically ill or at-risk patients, given its potential clinical impact [10].

The pathophysiological mechanisms involved in phosphorus homeostasis, their clinical relevance and the appropriate diagnostic approach for their interpretation in the context of PHPT are examined below.

### 3.1. Pathophysiology of Hypophosphatemia in Primary Hyperparathyroidism

Phosphorus represents approximately 1% of total body weight [11,12]. Approximately 85% is stored in bone as hydroxyapatite, ~14% is intracellular (as ATP, nucleotides, phospholipids), and ~1% circulates extracellularly as inorganic phosphate (Pi), the fraction measured clinically [11,12]. Circulating Pi is in equilibrium between its predominant ionic forms: HPO_4_^2−^ and H_2_PO_4_^−^; [13,14]. Its serum concentration is modulated by multiple factors, such as dietary intake, circadian rhythm, age and blood pH [13,14].

The main source of phosphorus is dietary intake, whether in organic form (e.g., vegetable phytates or animal proteins) or inorganic (e.g., phosphate salts in food additives) [11,12,13]. Dietary phosphorus is absorbed via two main intestinal mechanisms: passive paracellular absorption (65–80%), mediated by SLC9A3 also known as Na+/H+ exchanger (NHE)−3, and active transcellular transport via the sodium-dependent cotransporter SLC34A2, stimulated by calcitriol under conditions of low intake or high demand [12,15,16].

Renal phosphate handling is regulated by Na⁺/Pi type II cotransporters (IIa, IIb, IIc, encoded by SLC34A1, SLC34A2, SLC34A3) [13]. Under normal conditions, 85–95% of filtered phosphate is reabsorbed in the proximal tubule. In PHPT, the excess PTH directly inhibits the expression of the NaPi-IIa and NaPi-IIc cotransporters, reducing tubular reabsorption of phosphate and increasing its urinary excretion [12,13,14,17]. Resulting in sustained hypophosphatemia, even in the early stages of PHPT when serum calcium is still within the normal range.

This mechanism explains why hypophosphatemia can be an early biochemical finding in PHPT. In fact, its persistence over time, in the absence of other secondary causes (such as renal failure, vitamin D deficiency, digestive loss, or Fanconi syndrome), should raise suspicion of a hormonal cause, with PHPT being one of the main entities to consider [16].

FGF23, a phosphaturic hormone secreted by osteocytes and osteoblasts, may also contribute to hypophosphatemia, although its principal role is described in disorders such as oncogenic osteomalacia and hereditary rickets [18]. FGF23 downregulates the enzyme 1α-hydroxylase (1αOHase), reducing calcitriol synthesis and induces 24-hydroxylase, promoting its catabolism [19].

Experimental data suggest a bidirectional interaction between PTH and FGF-23: PTH can stimulate FGF-23 expression, while FGF-23 may downregulate PTH synthesis [20,21,22,23,24]. Clinical studies in PHPT have shown heterogeneous results. Early work suggested only modest elevations when renal function was preserved [24], whereas more recent cohorts demonstrated consistently higher levels that decrease after parathyroidectomy, particularly in patients with hypophosphatemia [18,25,26]. These findings support the view that elevated FGF-23 in PHPT is not merely a compensatory mechanism but rather represents a biochemical phenotype of more severe disease. In our patient, the markedly elevated FGF-23 levels, despite moderately preserved renal function, align better with this phenotype than with a purely adaptive response.

### 3.2. Clinical Relevance of Chronic Hypophosphatemia

The severity of hypophosphatemia carries important clinical implications. Severe hypophosphatemia can cause muscle weakness, bone pain, neuropathy, and cardiac dysfunction [7], although symptomatology also depends on patient age, comorbidities, and duration of deficiency [6,7].

Chronic hypophosphatemia is associated with a wide spectrum of clinical disorders, predominantly skeletal, as in the case of our patient, resulting from sustained defective bone mineralization mechanisms [17,27,28]. In the pediatric population, the manifestation of this disorder is as rickets [29,30], and in adults, it leads to osteomalacia [31,32], which manifests as diffuse bone pain, proximal muscle weakness, and a tendency for insufficiency fractures and pseudofractures, especially in weight-bearing bones [33,34,35]. These findings were evident in the presented case, where the patient exhibited multiple stress fractures involving the ribs, spine, and pelvis—hallmarks of impaired bone mineralization.

Furthermore, some studies demonstrate an association between hypophosphatemia and an increased risk of osteoporosis, kidney stones and poor outcomes in patients with PHPT (the latter especially in cases of moderate hypophosphatemia) [35,36].

### 3.3. Diagnostic Approach to Hypophosphatemia

Serum phosphate should ideally be measured fasting and in the morning due to circadian and postprandial variability [6]. Once hypophosphatemia is confirmed, acute reversible causes (e.g., alkalosis, refeeding, diabetic ketoacidosis, hungry bone syndrome) should be excluded [35]. Medication history is critical, given associations with agents such as intravenous iron, insulin, phosphate binders, cytotoxics, and antiretrovirals [9,18]. Pediatric cases often involve hereditary disorders, while adult cases are usually acquired (e.g., oncogenic osteomalacia, malabsorption, drug toxicity, Fanconi syndrome) [33,34]. Given the absence of an identifiable acute or iatrogenic cause, assessment of renal phosphate handling is essential. Tubular reabsorption of phosphate (TRP) or TmP/GFR can be calculated using serum and urine phosphate and creatinine; TRP ≥ 85% suggests extrarenal loss, whereas lower values indicate renal wasting [9,37]. Additional evaluation includes calcium, 25(OH)D, PTH, and FGF23 levels, tailored to suspected underlying causes [36,37,38,39].

In our patient, the differential diagnosis of hypophosphatemia performed exclusion of other causes of renal phosphate wasting. Elevated PTH and serum calcium suggested PHPT, but the absence of definitive findings on initial imaging prompted consideration of other, less common but clinically relevant causes of hypophosphatemia [38]. Despite the patient’s age and clinical presentation, genetic testing was performed to rule out other conditions such as autosomal dominant or X-linked hypophosphatemia rickets [38]. Fanconi syndrome was considered highly unlikely, given the absence of glucosuria, hypouricemia, aminoaciduria, or metabolic acidosis, together with normal calcitriol levels [38]. Once 18F-choline PET-CT became available at our centre, it was performed to confirm the presence of parathyroid lesions and to determine whether additional imaging was needed to exclude phosphaturic mesenchymal tumours producing FGF-23 (oncogenic osteomalacia) [38]. In such a scenario, 18F-FDG PET would have been considered, although other modalities such as Octreoscan, CT, or MRI are also employed in clinical practice [37].

### 3.4. Treatment of Hypophosphatemia and Therapeutic Implications in PHPT

Management depends on etiology, severity, chronicity, symptoms, calcium levels, and renal function [16]. Severe acute cases (<1 mg/dL) may require intravenous replacement [16]. In chronic hypophosphatemia, correction should address the underlying cause. In mild cases, increasing dietary phosphorus intake may be sufficient [16]. In PHPT, parathyroidectomy is the definitive option in patients with a surgical indication [1,2]. However, in asymptomatic forms or with a non-classical phenotype, hypophosphatemia could be a marker of hormonal activity, anticipating hypercalcemia or symptoms in some cases [16]. Current data show that patients with PHPT and hypophosphatemia more frequently present with higher PTH and calcium levels, lower vitamin D levels and a more severe clinical phenotype [16,36,37]. In subgroups where the classic criteria are not met (e.g., age <50 years, marked hypercalcemia), moderate hypophosphatemia has been suggested as a useful supporting criterion to guide the surgical decision, especially in contexts where a complete evaluation is not possible [16].

## 4. Conclusions

In summary, PHPT results from renal phosphate loss induced by the action of PTH on the proximal tubule. It may precede the development of hypercalcemia and represents a potentially useful biochemical marker for suspecting the disease in subclinical or non-classical stages.

Incorporating hypophosphatemia as a complementary criterion in the diagnostic and therapeutic approach could facilitate the early identification of PHPT and improve clinical decision-making, particularly in cases that do not meet classical surgical indications. Further studies are warranted to evaluate its role in risk stratification and its potential inclusion in future clinical guidelines.

## Figures and Tables

**Table 1:** Evolution of analytical parameters during the follow-up.

Evolution of Analytical Parameters			
	2015	2021	2023
Serum calcium	9.8 mg/dL	10.5 mg/dL	10.9 mg/dL
Serum phosphorus	0.8 mg/dL	1.5 mg/dL	1.7 mg/dL
Serum magnesium	1.4 mg/dL	1.3 mg/dL	-
Parathormone	169 pg/mL	164 pg/mL	121 pg/mL
25(OH)-vitamin D	28.5 mg/dL	31.9 mg/dL	30.2 mg/dL
eFGR	70 mL/min	60 mL/min	62 mL/min
FGF-23			1694 kRU/L
RTP	75%	-	56.2%

## Data Availability

The original contributions presented in this study are included in the article. Further inquiries can be directed to the corresponding authors.
